# Synergistic co-metabolism enhancing the crude oil degradation by *Acinetobacter oleivorans* DR1 and its metabolic potential

**DOI:** 10.1128/spectrum.03023-24

**Published:** 2025-05-21

**Authors:** Lairenjam Paikhomba Singha, Renuka Kumari, Keisam Malabika Singha, Piyush Pandey, Pratyoosh Shukla

**Affiliations:** 1 Enzyme Technology and Protein Bioinformatics Laboratory, School of Biotechnology, Institute of Science, Banaras Hindu University 30114, Varanasi, Uttar Pradesh, India; 2 Department of Microbiology, Central University of Rajasthan 206414, Ajmer, Rajasthan, India; 3 School of Life Science, Central University of Rajasthan 206414, Ajmer, Rajasthan, India; 4 Soil and Environmental Microbiology Laboratory, Department of Microbiology, Assam University 28686, Silchar, Assam, India; Gujarat Biotechnology University, Gandhinagar, Gujarat, India

**Keywords:** synergistic, co-metabolism, crude oil, biosurfactant, hydrocarbon, carbon catabolite repression (CCR)

## Abstract

Bacterial degradation of hydrocarbons during co-metabolism with glucose often resulted in variable degradation efficiency. This study explored the mechanistic understanding of the metabolic response during co-metabolism in *Acinetobacter oleivorans* DR1 using a metabolomics approach. We reported that glucose slowed the growth rate of DR1 with a 7-h lag phase in a combined media containing crude oil, glucose, and biosurfactant, yet glucose supplement enhanced the degradation rates by 31% for dodecane and 18% for triacontane compared to culture amended with crude oil and biosurfactant. This demonstrated that glucose may not be the preferred carbon and energy source, but it still significantly influences hydrocarbon metabolism through increased synthesis of fatty acids and low molecular weight organic acids (glutaric, citric, and tartaric acid, etc.). Principal component analysis revealed the distinct clusters of metabolites among the culture conditions, highlighting the minimal effect of biosurfactant. Our study reports the role of significant metabolites in crude oil culture, proving the gluconeogenesis capability of DR1. The pre-screening of the metabolic pathway concluded that trehalose was crucial in combating stress imposed during hydrocarbon degradation. Hence, *A. oleivorans* DR1 can be used to degrade hydrocarbons and has a pivotal role in synergistic co-metabolism during crude oil degradation.

**IMPORTANCE:**

In hydrocarbon-contaminated soil, the presence of easily metabolizable carbon sources can lead to carbon catabolite repression. This repression reduces the activity of hydrocarbon-degrading bacteria, slowing down the rate of bioremediation. In this study, the most robust yet underexplored tool—liquid chromatography-high resolution accurate mass spectrometry system—was used to study the metabolic response and functional state of A. oleivorans DR1 during the crude oil degradation in the presence or absence of either biosurfactant or glucose supplementation. Here, synergistic co-metabolism by DR1 for crude oil degradation is reported. DR1 preferred hydrocarbons over glucose since glucose was not readily utilizable due to lack of enzymes (e.g., glucokinase). However, the glucose enhanced the hydrocarbon degradation in DR1 through the high production of organic acids that reacted on hydrocarbon chains and underwent fatty acid synthesis. This study added *Acinetobacter oleivorans* DR1's strength towards hydrocarbon utilization and proposed it as an effective agent for bioremediation.

## INTRODUCTION

The demand for petroleum oil continues to increase worldwide, and it is expected to be 101 million barrels per day by 2030 (1, 2). The high demand has led to increased hydrocarbon contamination in terrestrial and marine environments, including agricultural lands ranging between 10^−3^ and 10^2^ g/kg of soil across the globe (3). Petroleum hydrocarbons (PHCs) are naturally occurring complex, heterogeneous mixtures primarily composed of hydrocarbons (aliphatic chains, aromatic, polyaromatic, and large complex compounds—asphaltenes and resins). Around 80% of crude oil is made up of saturated and aromatic hydrocarbons (4). Sites contaminated with oil are primarily affected by these compounds, yet n-alkanes, predominant in petroleum, can also be detected in pristine environments (5, 6). While microbial activity efficiently degrades the light fractions of PHCs, the heavier fractions that spill into soil pose challenges for degradation, adversely impacting ecosystems and organisms (7). Hydrocarbons exhibit lower bioavailability owing to their hydrophobic nature, which restricts their interactions with microorganisms and slows down degradation (8). The effectiveness of this process primarily depends on how each of these components, that is, microorganisms, oxygen, water-soluble minerals, and hydrocarbons, transfer and interact with each other (9).

In contaminated environments, biosurfactants are thought to be a viable method for more efficient hydrocarbon removal (10). These biosurfactants emulsify hydrocarbons and increase their solubility in the aqueous phase by reducing interfacial tension (11). Another significant hindrance in bioremediation processes is the phenomenon known as carbon catabolite suppression (CCR), which occurs when the presence of glucose prevents the utilization of secondary carbon sources (12). A major aspect of biodegradation is the selective breakdown of hydrocarbon compounds, even when simpler carbon sources like glucose are accessible. In some studies, the addition of glucose did not improve the degradation rate of polycyclic aromatic hydrocarbons (PAHs) (13, 14), while others reported increased degradation of PAHs and PHCs with glucose addition (15).

**Fig 1:**
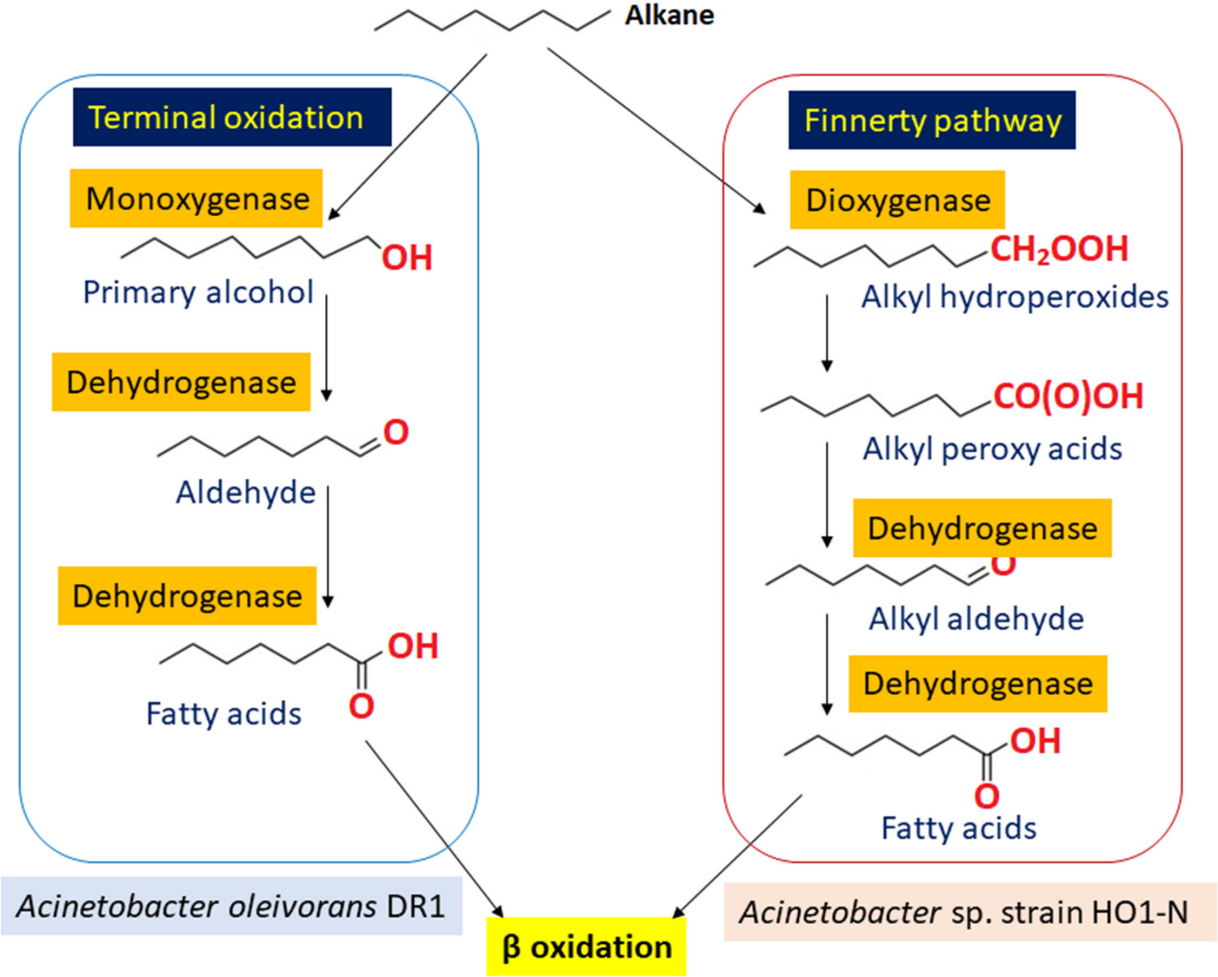
The common pathway of alkane oxidation in *Acinetobacter* species: terminal oxidation in *Acinetobacter oleivorans* DR1 and Finnerty pathway in *Acinetobacter* sp. strain HO1-N.

More than 200 microbial species have been reported for their capability of utilizing PHCs as their main source of carbon, energy, and metabolizing them into non-toxic carbon dioxide, water, and biomass (16). This metabolic pathway is observed in diverse bacterial species (16). Most notably, the genera include *Bacillus*, *Klebsiella*, *Pseudomonas, Proteus*, *Acinetobacter*, *Brevibacterium*, *Nocardia*, *Vibrio*, *Corynebacterium*, *Rhodotorula*, *Sporobolomyces*, *Aeromonas*, *Thiobacillus*, *Lactobacillus*, *Candida*, *Neurospora*, *Rhizopus*, *Mucor*, and *Trichoderma* (17). In aerobic conditions, oxygen is essential to initiate degradation, while sulfate or nitrite is required in anaerobic conditions (16). The breakdown of hydrocarbons, such as alkanes, begins with oxygenases that catalyze the introduction of oxygen atoms through various pathways in the presence of oxygen. In *Geobacillus thermodenitrificans*, the terminal oxidation of n-alkanes involves the attack on the terminal methyl group, converting it to a primary alcohol, which is then further oxidized into fatty acids by dehydrogenases. In bacteria like *Pseudomonas aeruginosa* and *Gordonia* sp*.*, alkane oxidation starts from the subterminal position, producing both primary and secondary alcohols or methyl acetone. The oxidation of acetone forms esters, which undergo hydroxylation by esterases, leading to the production of alcohols and fatty acids (16). Typically, the methyl group of n-alkanes is oxidized to form alcohols, which are then further oxidized to fatty acids by dehydrogenases (18). Singer and Finnerty described a unique pathway in *Acinetobacter* sp. strain HO1-N. Here, peroxy acids, hydroperoxides, and fatty acids are produced from the oxidation of long-chain alkanes. The pathway begins with dioxygenase catalyzing the initial step of n-alkane oxidation in *Acinetobacter* sp. (19–21). Cai et al. 21 outline two common alkane oxidation pathways in *Acinetobacter* sp., with the terminal oxidation pathway followed by *Acinetobacter oleivorans* DR1 in this study (Fig. 1) (21). The bacterial genera *Acinetobacter* are well known for their capability to break down hydrocarbons, particularly alkanes of various chain lengths. It is commonly detected in a broad array of sites contaminated with hydrocarbons such as soils, pristine habitats, and Antarctic marine sediments, underscoring its capacity for biodegradation of alkanes (22–24). *A. oleivorans* DR1 has been increasingly documented in environmental research within the *Acinetobacter* genus. This strain DR1 efficiently degrades aliphatic hydrocarbons, such as diesel fuel, partly by producing exopolysaccharides (EPSs) that facilitate the degradation process (25). Also, DR1 enhanced biofilm formation through increased EPS production in response to endogenous hydrogen peroxide (26). This strain had been examined for metabolic and stress responses during the biodegradation of n-alkanes. The Ack-Pta and glyoxylate shunt and pathway are likely crucial for metabolizing triacontane (C_30_ alkane) and safeguarding cells against oxidative stress induced by the degradation process (27).

In the present study, the growth kinetics of *A. oleivorans* DR1 in different culture conditions was examined to understand the effect of co-metabolism and biosurfactant on crude oil degradation and metabolism. Here, *A. oleivorans* DR1 was employed to understand the metabolic response during co-metabolism of hydrocarbons and glucose, since this strain has been studied for various magnitudes to understand the bioremediation capability. A metabolomics study of bacteria in varying culture conditions can help identify differences in metabolite production, providing insights into metabolic pathways. We employed a liquid chromatography-high-resolution accurate mass spectrometry system, a robust yet frequently underexplored tool for investigating the whole metabolic profile in microbial systems, allowing for real-time analysis of metabolic responses of *A. oleivorans* DR1 cultured in a minimal medium with or without biosurfactant supplementation, under the sole carbon sources of glucose or crude oil and their combination. This study primarily focused on a metabolomics approach to predict metabolic pathways and functional state, using the KEGG and MetaCyc databases alongside gene annotations from the genome of *A. oleivorans* DR1. Hence, the conclusion was drawn in this study as an initial validation to show that the metabolic route shifted due to the presence of different carbon sources during crude oil degradation. Also, this study describes that glucose may not be a preferable carbon and energy source in *A. oleivorans* DR1 but is also an important factor for the metabolism of other carbon sources like hydrocarbons. This process highlights the bacterium’s unique adaptation to utilize glucose for accelerating hydrocarbon breakdown in diverse environmental conditions.

## RESULTS

### Biosurfactant screening and characterization

*A. oleivorans* DR1 showed 50% emulsification at 144 h of incubation. Extracted biosurfactant was characterized by thin-layer chromatography (TLC) and FTIR. TLC was used to determine the nature of extracted biosurfactant. When the fully run TLC plate was submerged in different developing reagents, distinctive spots were seen. The spots' coloring in relation to the developing agent revealed information on the chemical composition of the spots (see Fig. S1 at https://github.com/Pratyoosh2025/Supplemental-Material--Synergistic-co-metabolism-by-Acinetobacter-oleivorans-DR1-.git). When treated with orcinol, brown spots test positive for rhamnose sugar, whereas yellow spots exhibit the lipid nature of the extracted biosurfactant when treated with bromothymol blue. As a result, the type of extracted biosurfactant was rhamnolipid. The functional group present in biosurfactants was recognized by using Fourier transform infrared (FTIR) spectroscopy). The biosurfactant produced by *A. oleivorans* DR1 showed peaks at 1,660 cm^−1^ (Ester groups in C–O stretching and bonded to fatty acids), 1,535 cm^−1^ (aromatic ring stretch), 1,403 cm^−1^ (C–H bending), 1,239 cm^−1^ (deformation and bending vibrations of –C–CH_2_ and –C–CH_3_ groups in the aliphatic chain), 3,417 cm^−1^ (representing the O–H stretching vibrations of amine or hydroxide groups), 1,063 cm^−1^ (C–O–C vibration in cyclic structure of carbohydrate), and 2,961 cm^−1^ (C–H stretching vibrations of alkyl (CH_2_–CH_3_) (see Fig. S2; Table S1 at https://github.com/Pratyoosh2025/Supplemental-Material--Synergistic-co-metabolism-by-Acinetobacter-oleivorans-DR1-.git).

### Growth of *A. oleivorans* DR1 under different carbon sources with or without biosurfactant

**Fig 2:**
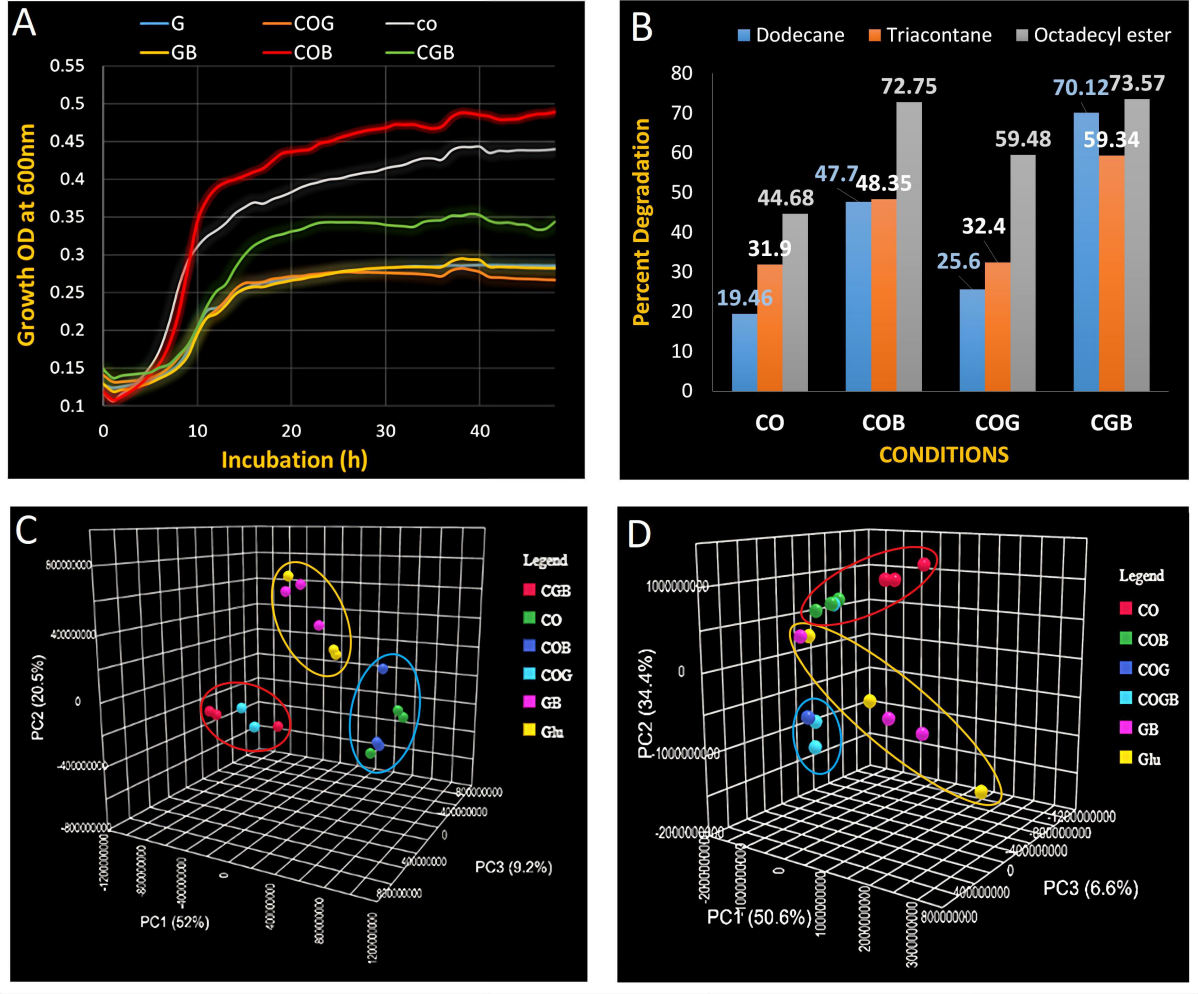
(A) Growth of *Acinetobacter oleivorans* DR1 at different carbon sources: G (glucose), COG (glucose and crude oil), CO (crude oil), GB (glucose and biosurfactant), COB (crude oil and biosurfactant). and CGB (glucose, crude oil and biosurfactant). (B) Percent degradation of crude oil components (dodecane, triacontane, and octadecyl ester). (C) PCA (negative mode). (D) PCA (positive mode). Partial least squares-discriminant analysis (PLS-DA) for the significant variation of metabolite production.

*A. oleivorans* DR1 is a well-known hydrocarbon-degrading gram-negative bacteria. This bacterium also produces rhamnolipid biosurfactant. In this study, the effect of biosurfactant and glucose on the growth of DR1 was observed in crude oil amended minimal medium. The growth rate was higher in minimal medium containing crude oil amended with biosurfactant than in the glucose amended minimal medium with or without the presence of biosurfactant (Fig. 2A). Glucose may not be the preferred carbon source, as its presence slowed down growth, resulting in a 7-h lag phase in all glucose-amended media (CGB, COG, GB, and G). In contrast, DR1 exhibited faster growth with a shorter lag phase of up to 3 hours in crude oil-based culture media (CO and crude oil and biosurfactant [COB]). The generation time during the exponential phase was 72 min in the crude oil and glucose-amended medium (CGB), whereas it was approximately 31 min in the crude oil-amended medium, where glucose was absent (COB).

### Effect of biosurfactant and glucose in crude oil degradation

The three alkanes—dodecane, triacontane, and octadecyl ester—were predominantly present in this type of crude oil. Therefore, these three alkanes were considered for the correlation of crude oil degradation among the given conditions. The degradation of three hydrocarbons—dodecane, triacontane, and octadecyl ester—was noticed in crude oil amended medium with or without the presence of biosurfactant and glucose. Most commonly, the presence of biosurfactants increases the absorption of hydrophobic compounds like hydrocarbons and thereby increases the rate of hydrocarbons—dodecane (59%), triacontane (34.6%), and octadecyl ester (38.6%) degradation higher than CO culture by the DR1 culture in COB (Fig. 2B; see Fig. S3 at https://github.com/Pratyoosh2025/Supplemental-Material--Synergistic-co-metabolism-by-Acinetobacter-oleivorans-DR1-.git). This bacterium possesses various enzymes, such as hydroxylases and oxygenases, involved in the metabolism and degradation of hydrocarbons (see Tables S2 to S7 at https://github.com/Pratyoosh2025/Supplemental-Material--Synergistic-co-metabolism-by-Acinetobacter-oleivorans-DR1-.git). By increasing the accessibility of hydrocarbons to these enzymes, biosurfactants can accelerate the degradation process, ultimately converting hydrocarbons into metabolites that bacteria can use for energy and growth.

The presence of glucose had more impact on the optimal utilization of crude oil and more degradation of crude oil components (Fig. 2B). The percent degradation rate of dodecane, triacontane, and octadecyl ester was significantly higher in CGB culture followed by COB, COG, and CO alone, respectively. In CGB, the presence of glucose showed 31% (dodecane) and 18% (triacontane) higher degradation rate than COB, while octadecyl ester showed similar degradation rates—72.75% and 73.57% in both the conditions—CGB and COB, respectively (both conditions showed 38–39% higher degradation of octadecyl ester than CO culture).

### Principal component analysis (PCA) of the metabolome of *A. oleivorans* DR1 under the culture conditions of different carbon sources

The variation of the metabolome in *A. oleivorans* DR1 under different culture conditions (CO, COB, and COG). COGB, GB, and Glu were visualized in PCA. Here, the variance of principal component (PC1 = 52%, PC2 = 20.5%, and PC3 = 9.2%) was described in negative ion detection. The statistically significant (*P* < 0.05) disperse metabolome was observed in CO culture from other culture conditions. The biosurfactant altered the metabolome when it was amended in the CO culture. Otherwise, there was no significant result of biosurfactant incorporation in other culture conditions, except it was observed in COB. There was less variance of the metabolome between the CGB and CG, thus forming close clusters. However, the clusters of CGB and CG, G and GB, and CO and COB were distantly separated from each other, indicating that the variation in the metabolome of DR1 in each culture condition (Fig. 2C). The similar variance of the metabolome was also visualized in positive ion detection. The less metabolome variation of DR1 in CGB and CG formed close clusters which were distantly separated from the clusters of COB and CO (Fig. 2D). Here, the biosurfactant had no role again in the variation of the metabolome of DR1. Instead, crude oil and glucose had shown to be responsible for the different metabolic flow.

### Partial least squares-discriminant analysis (PLS-DA) for the significant variation of metabolite production

The variables most responsible for separation in the PLS-DA were ranked in accordance with the variable importance in projection (VIP) scores. The VIP parameter was used to detect the most distinctive metabolites that distinguish the six culture conditions. The top 25 metabolites with VIP scores over 1.0 were highlighted (see Fig. S4 at https://github.com/Pratyoosh2025/Supplemental-Material--Synergistic-co-metabolism-by-Acinetobacter-oleivorans-DR1-.git). The metabolites identified as most discriminating between the six culture conditions were citric acid, trehalose, gluconic acid, and followed by others (see Fig. S4 at https://github.com/Pratyoosh2025/Supplemental-Material--Synergistic-co-metabolism-by-Acinetobacter-oleivorans-DR1-.git). The important identified metabolites in different culture conditions are provided in the supplementary file (see Table S2 at https://github.com/Pratyoosh2025/Supplemental-Material--Synergistic-co-metabolism-by-Acinetobacter-oleivorans-DR1-.git).

**Fig 3:**
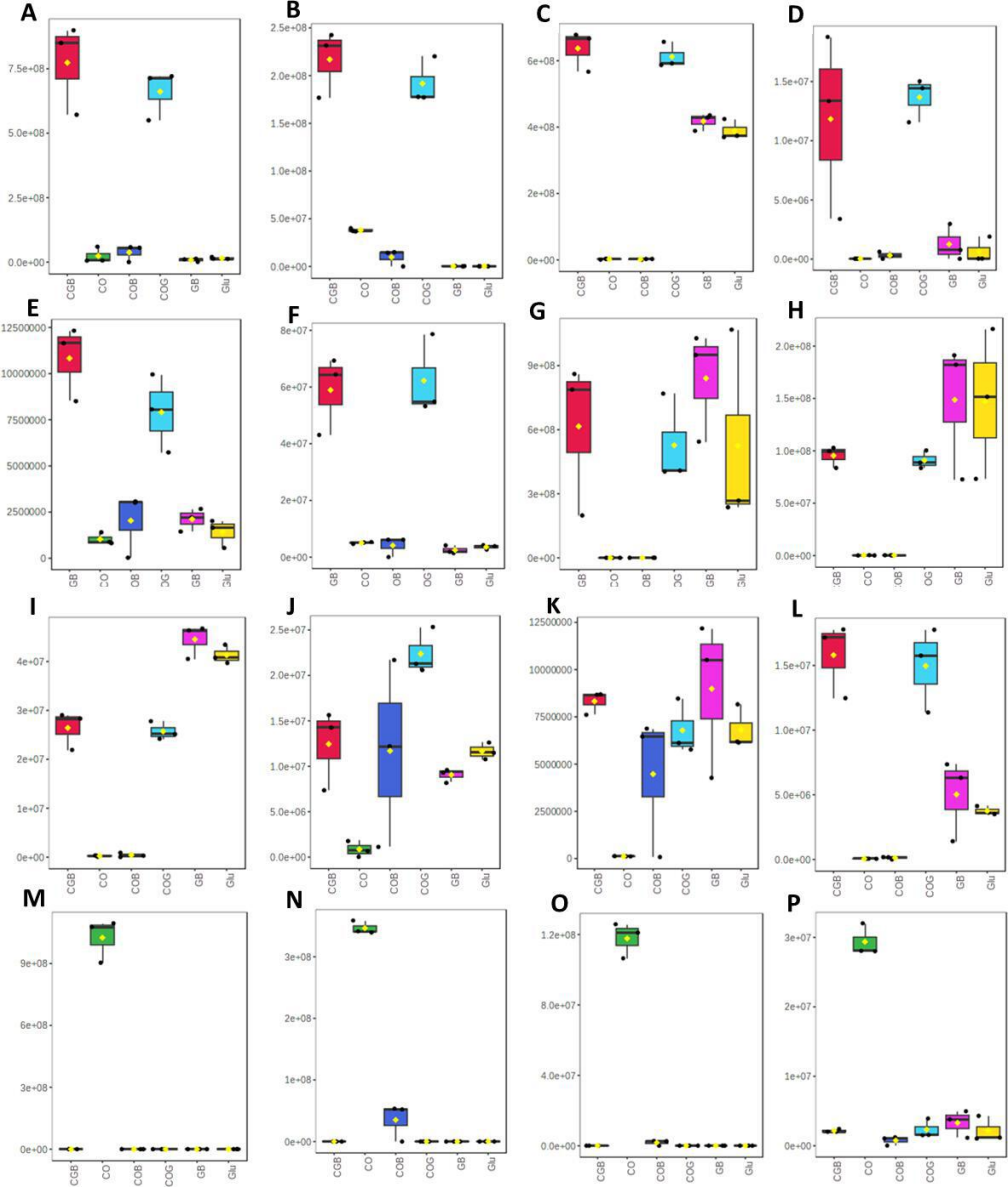
Box plot of highly differential metabolite accumulation in six different culture conditions—different carbon sources—G (glucose), COG (glucose and crude oil), CO (crude oil), GB (glucose and biosurfactant), COB (crude oil and biosurfactant), and CGB (glucose, crude oil and biosurfactant). (A) Citric acid, (B) Tartaric acid, (C) d-glucose, (D) Oleic acid, (E) Malic acid, (F) Furoic acid, (G) Gluconic acid, (H) Glutaric acid, (I) Alpha-ketoadipic acid, (J) Stearic acid, (K) 16-hydroxydecanoic acid, (L) d-glucopyranosyl, (M) Trehalose, (N) N-Acetyl-d-glucosamine 1-phosphate, (O) l-Glutathione oxidized, and (P) Succinic acid.

Biotin was highly accumulated in the crude oil culture, where it was negligible in other culture conditions. The other metabolites—l-glutathione oxidized, trehalose, glucose-6-phosphate, succinic acid, 2-C-methyl-d-erythritol-2,4-cyclopyrophosphate, 16-hydroxydecanoic acid (palmitic acid), N-acetyl-d-glucosamine 1-phosphate**,** FAD, ADP, GMP, TMP, and CMP—were significantly higher in CO culture. The higher accumulation of the metabolites—citric acid, tartaric acid, oleic acid, malic acid, furoic acid, d-glucopyranosyl—was detected in COG and CGB culture. The other metabolites—d-glucose, gluconic acid, glutatric acid, alpha-ketoglutaric acid, stearic acid, and so on—were also produced highly in glucose-containing cultures—CG, CGB, G, and GB—except CO and COB. The 16-hydroxydecanoic acid was also one of the common metabolites observed in all the culture conditions (Fig. 3).

### Gene annotation associated with metabolic pathways in *A. oleivorans* DR1

**Fig 4:**
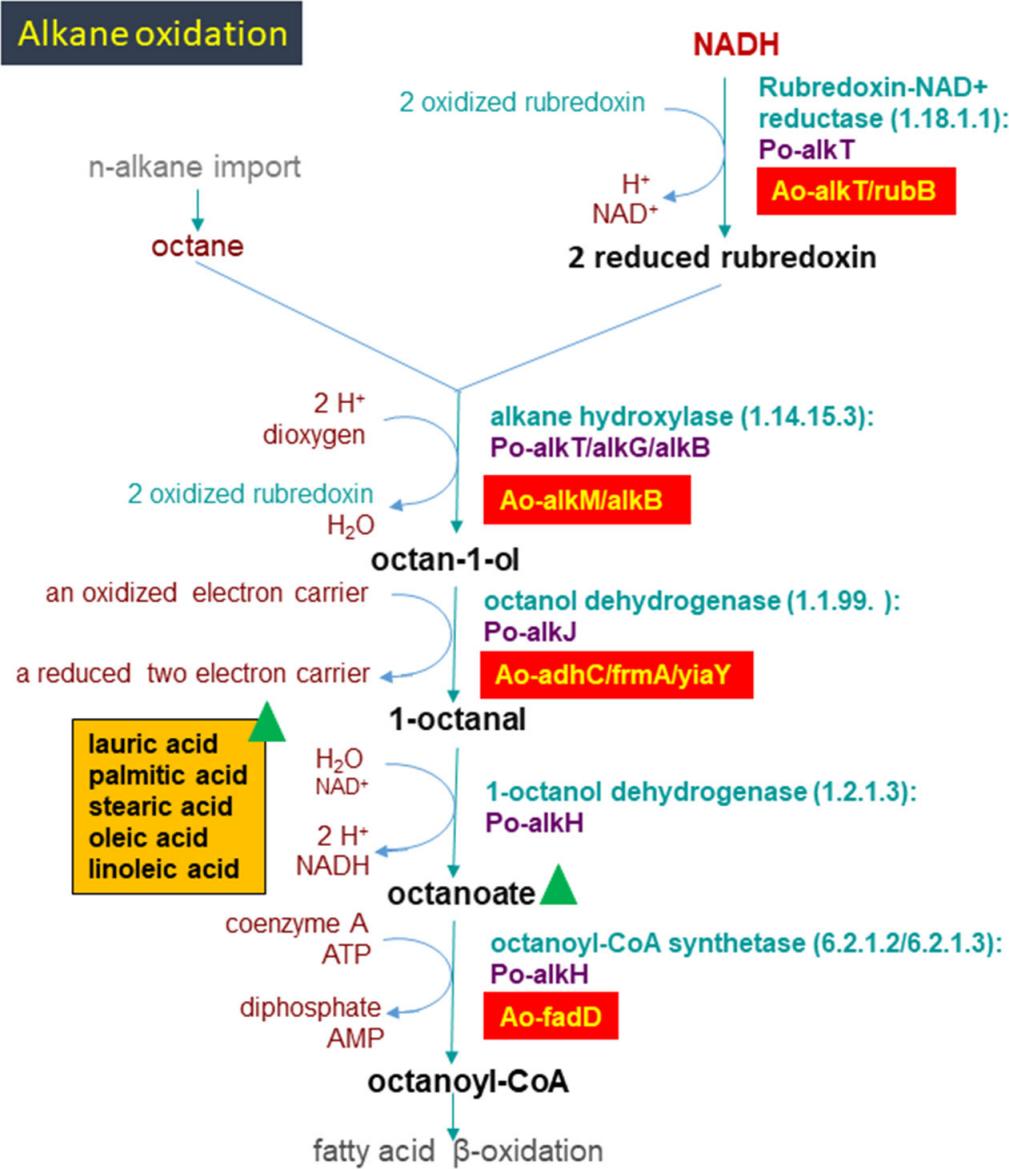
An illustrative depiction of the pathway describing the alkane oxidation (e.g., octane) by the common gram-negative bacterium. The genes in the genome of *A. oleivorans* DR1 involved are highlighted in red color. Fatty acids detected highly in the presence of glucose and crude oil metabolism in DR1 are highlighted in orange color. Ao, *A. oleivorans* DR1; Po, *Pseudomonas oleivorans*.

**Fig 5:**
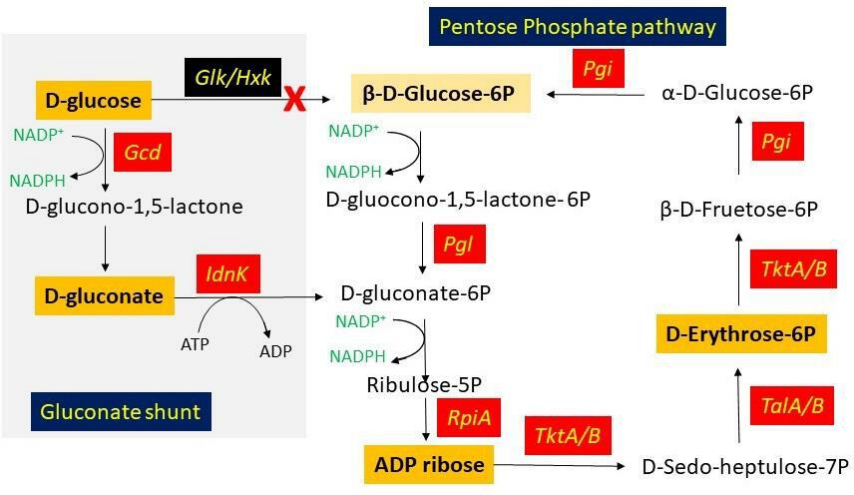
The gluconate shunt directs the glucose into the pentose phosphate pathway (PPP). Metabolites highlighted in bold with orange color have been detected in all the glucose-amended culture conditions. The genes *Gcd and IdnK* are the two genes responsible for the gluconate shunt. The other genes involved in the PPP are highlighted with red color. Glucokinase/hexokinase genes *Glk/Hxk* were not present in the genome of *Acinetobacter oleivornas* DR1, and therefore, no direct formation of d-glucose-6-phosphate from d-glucose.

The genome of *A. oleivorans* DR1 contains a high abundance of genes related to fatty acid metabolism (see Table S3 at https://github.com/Pratyoosh2025/Supplemental-Material--Synergistic-co-metabolism-by-Acinetobacter-oleivorans-DR1-.git). It also contains genes involved in hydrocarbon degradation, including *RubB*, *AlkT*, *AlkM*, *AdhC*, *FrmA*, *YiaY*, and *FadD*, which are linked to the alkane oxidation to fatty acids pathway. In addition, fatty acids such as linoleic acid, oleic acid, stearic acid, palmitic acid, and lauric acid were detected at high levels during glucose and crude oil metabolism in DR1 (Fig. 4). It also harbors all the genes required for glycolysis/gluconeogenesis and the pentose phosphate pathway (PPP), with the exception of *Glk*, which encodes glucokinase—an essential enzyme for the initiation of glycolysis that converts glucose to glucose-6-phosphate (see Fig. S7 at https://github.com/Pratyoosh2025/Supplemental-Material--Synergistic-co-metabolism-by-Acinetobacter-oleivorans-DR1-.git). As a result, DR1 predominantly utilizes the PPP when glucose is available in the culture conditions. DR1 possesses the genes *Gcd* and *IdnK*, which are involved in converting d-glucose to d-gluconate, initiating the PPP and producing d-gluconate-6-phosphate. Within the PPP, metabolites including ADP ribose, d-erythrose-6-phosphate, and glucose-6-phosphate were identified and linked to the genes *Pgl*, *RpiA*, *TktA*, *TktB*, *TalA*, and *TalB*, all of which are annotated in the genome of DR1 (Fig. 5). The *A. oleivorans* DR1 genome also includes genes for biotin synthesis, trehalose synthesis, and the mevalonate pathway. In addition, it harbors genes for benzoate degradation and other xenobiotic degradation pathways (see Table S5 at https://github.com/Pratyoosh2025/Supplemental-Material--Synergistic-co-metabolism-by-Acinetobacter-oleivorans-DR1-.git). The genes involved in biotin synthesis, such as *BioA*, *BioB*, *BioC*, *BioD*, *BioF*, *BioH*, *FabB*, *FabF*, *FabG*, *FabZ*, and *FabI*, have been annotated in the DR1 genome (see Table S8 at https://github.com/Pratyoosh2025/Supplemental-Material--Synergistic-co-metabolism-by-Acinetobacter-oleivorans-DR1-.git).

## DISCUSSION

The experiment was designed to assess the degradation rate of crude oil by *A. oleivorans* DR1 in the presence or absence of biosurfactant and glucose treatment. Hydrocarbons are generally hydrophobic (water-repellent) molecules and tend to form droplets or layers in an aqueous environment, making them less accessible to bacteria for metabolism. Biosurfactants, being amphiphilic molecules (having both hydrophilic and hydrophobic parts), can interact with hydrocarbons and water simultaneously (28). The emulsification of hydrocarbons by biosurfactants increased their bioavailability to DR1, facilitating their uptake and metabolism and thereby increasing the rapid growth in COB and CGB culture conditions (Fig. 2A). Here, we characterized that DR1 could synthesize its own biosurfactant rhamnolipid (see Fig. S1 and S2 at https://github.com/Pratyoosh2025/Supplemental-Material--Synergistic-co-metabolism-by-Acinetobacter-oleivorans-DR1-.git), yet incorporation of extracted biosurfactant in crude oil culture made them faster in growth and higher degradation rate than the CO culture alone. The growth curve of DR1 showed a short lag phase of up to 3 h in crude oil (both in CO and COB) culture medium, while the lag phase was 7 h in other glucose-amended medium (CGB, COG, GB, and G). Jung et al. 29 showed the inability of *A. oleivorans* DR1 for glucose metabolism, as the key genes for glycolysis, such as glucokinase, 6-phosphofructokinase, pyruvate kinase, or the Glc, Lac, Man, and Fru family phosphotransferase system, were lacking in the genome of DR1 (29). This may be the reason why DR1 showed the slower growth rate in G and GB and also because of the long lag phase due to the co-metabolism effect between glucose and crude, as in the case of CGB and COG. Moreover, the glucose content was 36% higher in the crude oil and glucose culture condition compared to the culture grown on glucose alone after 48 h of incubation. This indicates that DR1 utilized less glucose in the presence of hydrocarbons in the medium. In one of the studies, *Pseudomonas putida* CSV86 exhibited a diauxic growth pattern, with a gradual decline in glucose concentration during the second log phase and took an 8 h lag phase when the two carbon sources (benzoate and glucose) were given and proved the preference of aromatic hydrocarbon over glucose (12). The activity of catechol-1,2-dioxygenase, an important enzyme in the meta-cleavage pathway of benzoate degradation, was higher during the first log phase (10 h), while the activity of the glucose-metabolizing enzyme zwf (glucose-6-phosphate dehydrogenase) was significantly lower. In contrast, zwf activity increased markedly during the second log phase (20 h). Similarly, the unique ability of *A. oleivorans* DR1 to preferentially grow and metabolize hydrocarbons over glucose, as well as co-metabolize them to produce organic acids and fatty acids, makes this strain an excellent candidate for effective bioremediation of aromatics, even in the presence of simple carbon sources. This adaptability enables it to bypass carbon catabolite repression (12). The addition of biosurfactant increased the absorption of hydrophobic compounds like hydrocarbons and thereby increased the rate of hydrocarbons—dodecane (59%), triacontane (34.6%), octadecyl ester (38.6%) degradation higher than CO culture by the DR1 culture in COB (Fig. 2B). Similarly, the maximum degradation of phenanthrene by *Klebsiella* sp. was achieved in the rhamnolipid-containing medium (30). The addition of biosurfactants from *Pseudozyma* spp. to the culture broth improved the degradation of C_10_-C_24_ alkanes by *Pseudomonas putida* by approximately 46% (31). In an additional study, the efficacy of degradation of PAHs attained 86.5% after the incorporation of biosurfactants, compared to 57% in systems without biosurfactants or improvements of nutrient (32). Zeng et al. demonstrated that monorhamnolipids improved biodegradation of hexadecane by *Candida tropicalis*, possibly due to changes in cell surface properties (33). Additionally, the impact of rhamnolipid accumulation on phenanthrene (PHE) biodegradation by *Sphingomonas* sp. GF2B isolated from farmland soil was examined by Pei et al. 34. The unaltered strain exhibited a high phenanthrene degradation capability (83.6% mineralization), whereas rhamnolipid addition increased the biodegradation rate to 99.5% (34).

The ability of hydrocarbon (C_12_-C_30_ alkane) metabolism by *A. oleivorans* DR1 and its responsible genes such as *alkB1* and *alkB2* has been reported (27). In this study, the enhanced degradation of dodecane, triacontane, and octadecyl ester in glucose-amended medium—CGB and COG—was unexpected as it was contrary to growth (Fig. 2A and B). A native strain of *Pseudomonas* sp. WDE11, known for its ability to degrade polyaromatic hydrocarbons, effectively reduced 1% petroleum crude oil (PCO) in seawater when supplemented with glucose (1.0 g/L) (35). *Acinetobacter baumannii* OCB1, an aquatic environment isolate, demonstrated a 69.69% degradation of C8-C14 hydrocarbons in PCO contaminated with glucose 1.0 g/L. According to Ali Khan et al., improved nutrient accessibility and obtainability for microbial strains capable of utilization of hydrocarbon and production of biosurfactant lead to increased production of biosurfactant and, consequently, enhanced rates of bioremediation of hydrocarbons (36).

In CO culture conditions, the highly accumulated metabolite 16 hexadecanoic acid was the direct product of hydrocarbon degradation. This fatty acid was further utilized for ATP generation through β-oxidation. Liu et al. 37 described the mechanism of degradation of alkanes. When exposed to alkane, cells of *Gordonia sihwaniensis* triggered the secretion of abundant biosurfactants, effectively emulsifying large alkane particles into smaller ones. These alkanes were then transported across the cell membrane via vesicular transport, accompanied by ATP consumption. Inside the cell, n-hexadecane underwent a sequential conversion process catalyzed by monooxygenase, yielding n-hexadecyl alcohol, n-hexadecyl aldehyde, and n-hexadecanoic acid. These products subsequently entered the tricarboxylic acid cycle, generating numerous low-molecular-weight organic acids. The entire degradation process predominantly occurred intracellularly, highlighting the efficient bioremediation potential of *G. sihwaniensis* (37). The glutathione was highly produced in CO culture only to combat the toxic compound accumulation during the degradation of CO components, thus reducing stress. ROS stress is generated in *A. oleivorans* DR1 during growth on alkanes (26). Overall, l-glutathione oxidation to GSSG is an important aspect of cellular defense and detoxification processes in hydrocarbon metabolism. It serves to neutralize ROS, detoxify harmful hydrocarbon metabolites, and maintain cellular redox balance, thereby protecting cells from oxidative stress and damage associated with hydrocarbon exposure.

**Fig 6:**
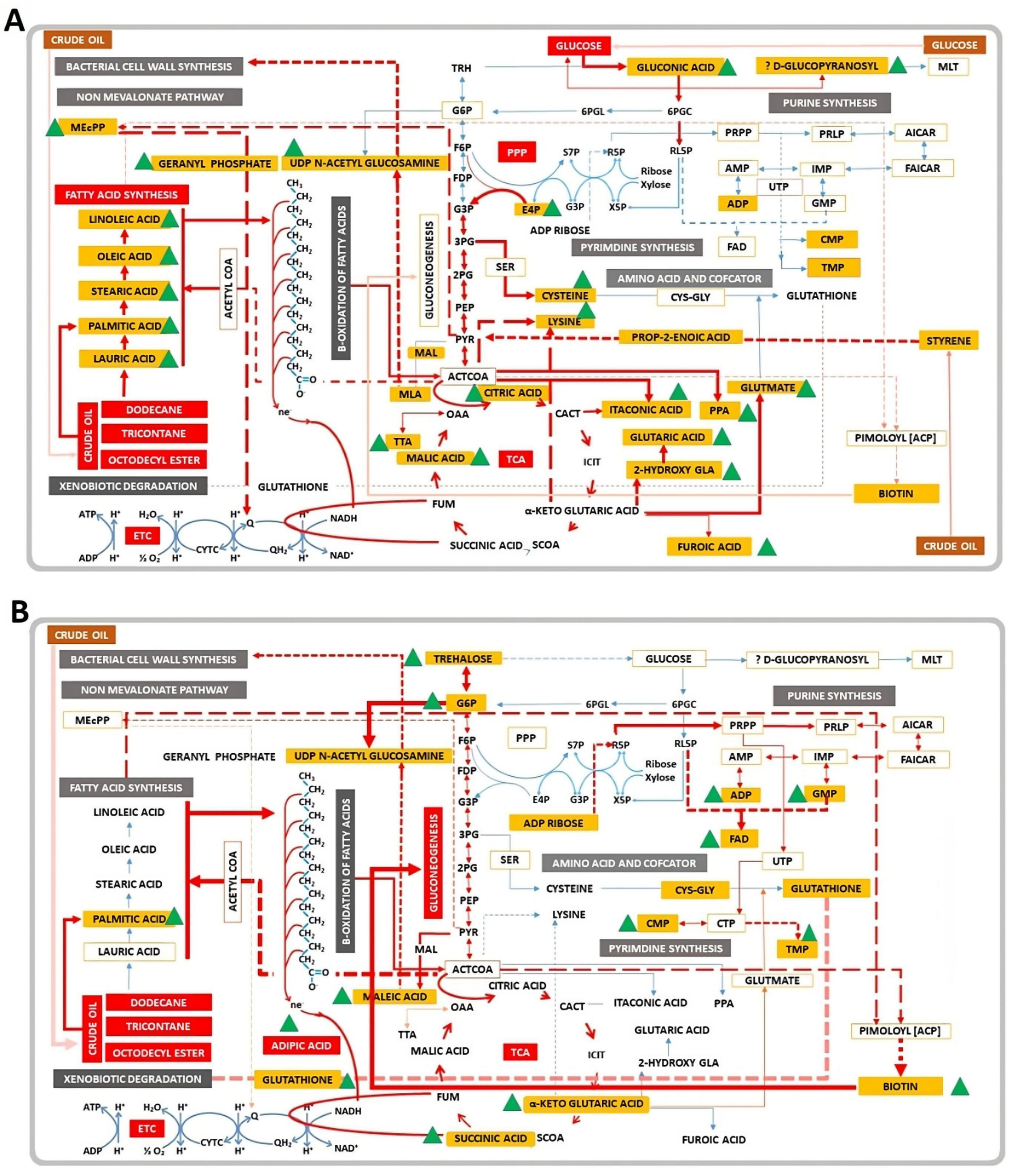
Top accumulated metabolites were closely associated with KEGG pathways. (A) The detected metabolites interlinked between pentose phosphate pathway, TCA cycle, and fatty acid synthesis in CGB specifically. (B) The detected metabolites interlinked between gluconeogenesis, β-oxidation of fatty acids, glutathione synthesis, and trehalose synthesis pathways. The isosceles triangle filled with green color is marked as high production metabolites.

Again, the presence of significant amounts of trehalose, glucose 6-phosphate, and biotin in CO culture described that DR1 had to undergo gluconeogenesis pathways (Fig. 6B). Glucose 6-phosphate is the metabolite which can be produced only through gluconeogenesis rather than glycolysis since it does not harbor glucokinase in *A. oleivorans* DR1. A glycosidic linkage joins the two d-glucose molecules and forms trehalose (38). It is synthesized by the TPS/TPP pathway involving two enzymes—trehalose 6-phosphate synthase (TPS or *OtsA*) and trehalose 6-phosphatase (TPP or *OtsB*) that can change to glucose 6-phosphate and UDP glucose to trehalose (38). Trehalose synthesis and its accumulation were beneficial to *A. oleivorans* DR1 because trehalose might protect from oxidative stress induced by hydrocarbon metabolism. Piazza et al. 39 reported that impairing trehalose biosynthesis in *Xanthomonas citri* lowered the bacteria’s ability to withstand oxidative stress induced by H_2_O_2_ (39). Trehalose lies in the ability to reduce the damage to biomolecules via a series of mechanisms, interacts with fatty acids, preventing oxidative damage and the formation of H_2_O_2_ (40). The synthesis of trehalose significantly by DR1 portrayed another advantage that this bacterium can be efficient root-colonizing bacteria if augmented in the plant rhizosphere. For instance, root nodule colonizing rhizobacteria—*Rhizobium leguminosarum*, *Sinorhizobium meliloti*, and *Bradyrhizobium japonicum*—produced trehalose in high amounts that led to increased resistance to osmotic stress imposed by plants, protected and enhanced colonization (41–43). Gluconeogenesis that occurred in DR1 during hydrocarbon degradation could be confirmed by the high accumulation of biotin in the CO condition. Biotin, also known as vitamin H or B7, serves as a vital cofactor for biotin-dependent enzymes such as carboxylases, decarboxylases, and transcarboxylases. These enzymes play crucial roles in intermediary metabolism, such as gluconeogenesis, metabolism of amino acids, and synthesis of fatty acids (44). The high biotin accumulation in the CO condition was needed by DR1 for regulating gluconeogenesis. Crude oil degradation initiated as described above, and gluconeogenesis simultaneously occurred during the degradation process to synthesize trehalose. Both trehalose and hydrocarbons can serve as energy sources for organisms. Trehalose can be broken down into glucose, which can then enter metabolic pathways such as the PPP in DR1 as described below. Similarly, hydrocarbons stored in fats and oils can be broken down through processes like beta-oxidation to generate energy in the form of ATP.

In this study, we also observed that the hydrocarbons were preferable over glucose, even though DR1 has the complete genes for the PPP. The glucose level was higher in CGB, followed by COG than G and GB, whereas higher hydrocarbon degradation was observed in CGB (Fig. 6A). Since *A. oleivorans* DR1 lacks the gene coding for glucokinase, the conversion of glucose to glucose 6-phosphate was not detected. However, gluconic acid was significantly observed in all glucose-amended media. This can be attributed to the presence of the genes *Gcd* and *IdnK*, which encode glucose dehydrogenase and gluconate kinase, respectively (Fig. 5). These enzymes facilitate the conversion of glucose to gluconate (gluconic acid) via d-glucono-1,5-lactone in the PPP. This process is similar to an alternative pathway known as the gluconate shunt, proposed in *Schizosaccharomyces pombe*, which also utilizes the genes *Gcd1* and *Idn1*. This pathway directs glucose to undergo the PPP, bypassing hexokinase or glucokinase and the rate-limiting enzyme glucose-6-phosphate dehydrogenase (45). Wushensky et al. 46 employed stable isotope-assisted metabolomics profiling and metabolic flux analysis to provide a high-resolution understanding of the diverse catabolic pathways involved in carbohydrate metabolism in *Bacillus megaterium*. Their findings also confirmed that glucose metabolism via the gluconate shunt proceeds through the PPP (46). Gluconic acid is a significant natural acid produced moderately through the straightforward oxidation of glucose (47). As gluconic acid accumulates due to the oxidation of glucose, it lowers the pH of the medium. This acidic environment facilitates the entry of gluconic acid through the cell wall, after which it proceeds to enter the PPP for further metabolism (48). The bacterial isolates—*Stenotrophomonas* sp. MAL1 and *Arthrobacter* sp. MAL3 exhibited enhanced high molecular weight polyaromatic hydrocarbons with the addition of low molecular weight organic acids such as citric acid, malic acid, and oxalic acid (49). The presence of glutaric acid significantly enhanced the bioconversion rate of hexadecane to 1-hexadecanol, accelerating the initial enzymatic degradation step. This finding elucidates, for the first time, the mechanism by which glutaric acid promotes hexadecane degradation by a strain of *P. aeruginosa* (50). Similarly, the addition of glucose resulted in increased production of organic acids such as citric acid, malic acid, itaconic acid, glutaric acid, hydroxyglutaric acid, furoic acid, maleic acid, kojic acid, ascorbic acid, and tartaric acid significantly in CGB and COG than CO and COB. Here, we propose the mechanism how *A. oleivorans* DR1 used glucose to produce organic acids which played an important role in enhanced utilization of hydrocarbon (Fig. 2B and 3). The key genes (*AlkB, AlkT,* and *RubB*) involved in the alkane oxidation pathway were identified in the genome of *A. oleivorans* DR1 and compared with the known alkane (e.g., octane) oxidation described in *Pseudomonas oleovorans* (Fig. 4). The capacity to use hydrocarbons as the sole carbon and energy source is very common and not restricted to any particular group of microorganisms. Examples were found in a wide range of prokaryotic and eukaryotic genera including gram-negative and gram-positive bacteria, yeasts, fungi, and even some achlorophyllous algae. One of these organisms is the gram-negative bacterium Pseudomonas oleovorans (Po), which can grow on intermediate chain length n-alkanes (C_6_ to C_12_). In this pathway, the alkane hydroxylase system is the main functional protein carried in a plasmid known as OCT plasmid (51). The alkane hydroxylase system comprises three key components: NADH-dependent rubredoxin-NAD(+) reductase uses rubredoxin 2 to transfer electrons from NADH to alkane 1-monooxygenase, which catalyzes the incorporation of molecular oxygen into the terminal carbon atom of hydrocarbons (50), resulting in the formation of primary alcohols (51). The octan-1-ol undergoes further catabolism through the action of dehydrogenases and acyl-CoA synthetase to synthesize octanoyl-CoA as the final end product, then the β-oxidation cycle, serving as a carbon and energy source (52). The elevated transcription levels of genes encoding rubredoxin reductase and alkane hydroxylase were also reported in *Acinetobacter* sp., which were involved in hydrocarbon degradation in the oil reservoir (52). This alkane degradation pathway aligns with other terminal oxidation pathways found in bacteria like *Alcanivorax borkumensis*, *P. putida*, and *G. thermodenitrificans*, where hexadecane is converted to hexadecanoic acid for β-oxidation (53). In some hydrocarbonoclastic bacteria, such as *P. aeruginosa* and *Gordonia* sp., subterminal oxidation occurs, where hexadecane is oxidized to 2-hexadecanol, then processed into tetradecyl acetate before β-oxidation. This study contrasts with the Finnerty pathway in *Acinetobacter* sp., where dioxygenase converts hexadecane to hexadecaneperoxoic acid, which is then oxidized to hexadecanoic acid for β-oxidation (53). The high accumulation of fatty acids—lauric acid, palmitic acid, oleic acid, stearic acid, and linoleic acid—was observed during alkane degradation. Marine microbes also showed fatty acids as intermediate compounds during alkane degradation (54). These fatty acids, such as palmitic acid and oleic acid, also assisted hydrocarbons degradation in *Bacillus cereus* ND1 (55). Similarly, the high accumulation of these types of hydroxylated fatty acids was also observed during the degradation of n-alkanes by the three strains—*Novosphingobium* sp. S1, *Gordonia amicalis* S2, and *Gordonia terrae* S5, and suggested that these fatty acids can be used as a potential indicator for the degradation of hydrocarbons (56).

Another key consideration for biosurfactant treatment, despite being produced by DR1 itself, was that biosurfactants may exhibit toxicity to cells when added to the medium beyond a threshold level, disrupting the cell membrane upon interaction with lipid components (57). Potowary et al. reported that the application of rhamnolipid at 2 g/L resulted in lower TPH degradation compared to degradation at 1.5 g/L, likely due to the potential toxic effects of biosurfactants beyond their threshold levels (58). The incorporation of biosurfactants in the degradation of crude oil increased efficiency by 30% compared to using *Ochrobactrum intermedium* alone for petroleum sludge. This indicates that adding biosurfactants significantly benefited the degradation process (59). The *A. oleivorans* DR1 is a well-known alkane degrader and is also capable of producing biosurfactants. Despite the production of biosurfactants, this strain degraded alkanes more effectively when both biosurfactants and glucose were provided alongside crude oil in the initial culture phase. This experiment has confirmed that the biosurfactant (1 mg/mL) increased the growth rate and enhanced the crude oil degradation rate by *A. oleivorans* DR1. This study will be valuable for metabolic engineering aimed at increasing biosurfactant production in *A. oleivorans* DR1 and will assist in designing a medium specifically for DR1 in industrial applications and bioformulations for wastewater treatment plants.

Surfactants are in high demand globally; however, the use of synthetic surfactants can negatively impact human health and disrupt ecosystems due to their non-biodegradable nature. This highlights the need to prioritize naturally occurring green alternatives. Microbial biosurfactants offer a solution, as they are eco-friendly and biodegradable. They possess various beneficial properties, including heterogeneity, substrate specificity, and biodegradability, which have attracted significant research interest (28). These characteristics have spurred efforts for their large-scale production worldwide. The biosurfactant produced by *Pseudomonas cepacia* CCT6659 showed promise for biological remediation of soils. In trials with soil contaminated by hydrophobic organic matter, the combination of an indigenous consortium and the biosurfactant achieved a 95% reduction in contaminants within 35–60 days (60). In addition, biosurfactants are often utilized for soil remediation, boosting nutrient content, and serving as biocides aimed at bacteria. They enhance the bioavailability of pesticides, which speeds up the breakdown of contaminants in sediments and soil (61). *Acinetobacter venetianus* RAG-1 efficiently degrades three types of crude oil, showcasing strong emulsification activity and cell surface hydrophobicity, as well as broad environmental tolerance. It can utilize a variety of alkane substrates (C10–C38) through three alkane hydroxylases (AlkMa, AlkMb, and AlmA) (62). Similarly, *A. oleivorans* DR1 features an alkane hydroxylase system, and like the degradation capabilities of RAG-1, along with an engineered consortia strategy, indicate its potential for microbial biodegradation applications.

This study concluded that biosurfactant treatment (1 mg/mL) increased the growth of *A. oleivorans* DR1 and enhanced degradation of crude oil (dodecane, triacontane, and octadecyl ester). The metabolomics study of *A. oleivorans* DR1 revealed that glucose had a substantial role in the deviation of metabolic production during crude oil utilization and induced degradation rates higher when combined with biosurfactant. *A. oleivorans DR1* has an alkane hydroxylase system that allows it to tailor its metabolic response to different carbon sources, along with notable biosurfactant production. This study provides valuable insights into the mechanisms through which *A. oleivorans* DR1 can facilitate the bioremediation of hydrocarbons utilizing non-preferred glucose. The study refers to a key finding that *A. oleivorans* DR1 did not undergo glycolysis (EMP); rather, it preferred the gluconate shunt, which directed the PPP for the best utilization of glucose, fueling hydrocarbons degradation more easily via low molecular weight organic acid and fatty acid synthesis. By utilizing glucose through the gluconate shunt pathway, hydrocarbon-degrading bacteria may enhance their metabolic efficiency, especially in environments where hydrocarbons serve as the primary energy source, but glucose offers an additional or alternative supply of energy. We suggest that this pathway enables the organism to adapt and thrive in nutrient-limited or fluctuating conditions. The study finally deciphers the synergistic co-metabolism of *A. oleivorans* DR1, which in turn enhanced the crude oil degradation. This ability makes it a strong candidate for large-scale implementation in wastewater treatment plants. Future studies could explore scaling up or assessing the process under more variable conditions to better simulate natural environments.

## MATERIALS AND METHODS

### Chemicals, bacterial strain, and conditions of growth

*A. oleivorans* DR1, a diesel degrader, previously characterized for diesel degradation was used in this study (22). This strain *A. oleivorans* DR1 (MCC 2291) was obtained from the NCCS Pune, India. The strain DR1 was maintained using Luria Bertani Agar and stored in glycerol stock at −20°C with regular sub-culturing. The strain DR1 was initially grown on Bushnell Haas Agar amended with different carbon sources—crude oil (0.1%), crude oil and glucose (0.1%)—and incubated at 30°C kept for 72 h. Crude oil used in laboratory experiments was obtained from Srikona ONGC in Silchar, Assam, India. Hexane and ethyl acetate and hexane were collected from Sigma-Aldrich, and phosphate-buffered saline was provided by Hi-media, nutrient broth (NB), Luria Bertani Broth, and Bushnell Haas Broth in India.

### Screening for biosurfactant production and its characterization

The fresh bacterial culture at the exponential phase was transferred and grown in NB and incubated at 30°C under shaking conditions (140 rpm). NB is widely used in growth studies and biosurfactant production research, as reported in several studies (36, 63). The bacterial supernatant was collected every 24 h till 168 h, from a culture grown in NB. The supernatant was collected by centrifuging the bacterial culture for 10 min at 12,000 × *g*. An equal volume (1:1 vol/vol) of the cell-free culture supernatant and diesel oil was mixed in a test tube and vortexed for 10 min. The resulting emulsion was allowed to stabilize for 24 h under dark conditions. The emulsification index was then calculated using the following formula:

% of E24 = *H*_emulsion_**/***H*_total_ × 100

where *H*_emulsion_ is the emulsion’s height, and *H*_total_ is the liquid’s overall height (64).

### Extraction and characterization of biosurfactant

The same culture condition was followed for the extraction of biosurfactant from the bacterial culture as mentioned above, and the 100 mL culture was taken for the crude extraction of biosurfactant. A crude extract was obtained utilizing an acid precipitation and the solvent extraction procedure with slight modifications of Abbasi et al. (65). The maximum emulsification was noticed at 144 h of incubation during the screening of biosurfactant. Therefore, the cell-free supernatant was extracted from the culture broth at 144 h of incubation by centrifuging at 4°C for 10 min at 10,000 × *g*. The supernatant was treated with HCl 6 N to acidify it to a pH of 2. It was then kept at 4°C overnight before being extracted with chloroform and ethyl acetate in a ratio of 2:1, using a separatory funnel. The extract, a thick brown substance, was prepared for TLC and FTIR spectroscopy. For TLC, a solvent composition of 65:15:2 chloroform, methanol, and water was used. A 0.19% solution of orcinol and bromothymol blue was utilized to form spots on the TLC plate (64). *Alcaligenes faecalis* BDB4 (as positive control) was used to compare with DR1 on TLC plate only, since BDB4 was one of the bacterial strains which produced biosurfactant significantly (see Fig. S1 at https://github.com/Pratyoosh2025/Supplemental-Material--Synergistic-co-metabolism-by-Acinetobacter-oleivorans-DR1-.git). FTIR was used to detect the various bonds and functional groups retained in the biosurfactants by infrared spectrum analysis. The lines of spectral activity were captured between 4,000 and 400 cm^−1^. Using an infrared absorption frequencies database, the peaks were examined (64).

### Bacterial growth response under varying conditions in crude oil- and glucose-amended culture media

The fresh culture of *A. oleivorans* DR1 at exponential phase was collected and washed twice with saline water; subsequently, centrifugation was performed for 10 min at 10,000 rpm. The bacterium was inoculated with initial OD approximately 0.15 at 600 nm in BHB amended with different carbon sources in the presence or absence of biosurfactant (1 mg/mL). The growth conditions were—glucose (G, crude oil+glucose (COG), crude oil (CO), glucose + biosurfactant (GB), crude oil + glucose + biosurfactant (CGB), and crude oil + biosurfactant (COB). The crude oil (0.1%) and glucose (0.1%) were added in all the respective conditions. The freshly grown DR1 was inoculated in each condition and incubated in a shaker incubator (180 rpm) at 30°C for 48 h. To draw the bacterial growth curve, the similar conditions were applied simultaneously using the FlexA-200 Microplate Reader at 30°C for 48 h and growth OD (600 nm) was taken at every hour of interval.

### Gas chromatography-mass spectrometry (GC-MS) analysis for degradation of hydrocarbons in *in-vitro* condition

For the extraction procedure, the pre-established culture conditions were maintained for 48 h at 30°C in a rotary incubation shaker at 180 rpm to carry out the mixed crude oil treatments. Following these procedures, the whole contents of the individual sample were utilized for extraction. The residual crude oil was extracted in the organic phase during liquid-liquid extraction with ethyl acetate (1:1) and repeated two to three times. Subsequently, it was passed through Na_2_SO_4_ and evaporated via rotary evaporator for concentration. After dissolving in appropriate solvents, the quantitative estimation of crude oil degradation was performed using GC-MS (66). The GC-MS (GC-MSQP 2010 [SHIMADZU]) analysis utilized TR-35MS capillary column (30 m × 0.25 mm), with the thickness of the film 0.25 µm. Helium was employed as a carrier gas, where the rate of flow of the column was 1.51 mL/min (at 105 kPa). A 1 µL aliquot of the extract was inserted, and the temperature of the column was adjusted for 2 min at 70°C, before increasing at a rate of 10°C/min to 200°C and kept for 2 min. Afterward, the temperature was raised to 240°C for 2 min maintained at a rate of 5°C/min, subsequently by an upsurge to 300°C at a rate of 35°C/min for 2 min a rate of 35°C/min (scan range: 40–1,000 *m/z*). At 290°C, the transfer line and mass spectrometer were maintained. The data were compared in the mass spectra library system of inbuilt standard (NIST-05 and Wiley-8) of the GC-MS (67). The three alkanes—dodecane, triacontane, and octadecyl ester—were predominantly present in this type of crude oil. Therefore, these three alkanes were considered for the correlation of crude oil degradation among the given conditions. The percent degradation of hydrocarbons was calculated by the formula (68).

Degradation (%) = (C − T)/ C × 100

where C—Control (0.1% crude oil amended medium without bacterial inoculation) and T—Treatment (0.1% crude oil amended medium with bacterial inoculation).

### Metabolomics study of the bacterial culture

The sample was prepared from the 48 h old culture of *A. oleivorans* DR1 grown at different conditions as previously described. The bacterial pellet was collected after centrifugation at 12,000 × *g* for 10 min and washed with saline water. The cell pellet was again suspended in methanol and subjected to ultrasonication (~20 kHz) for 10 min. The methanol extract was vacuum dried, followed by the addition of 1 mL of mass spectrometry grade water to the sample. To each optimized sample, 1.5 mL of solvent (methanol:HPLC grade H_2_O, 80:20) was added for HR-MS analysis. The mixture was homogenized for 30 min at 750 rpm using an Eppendorf Thermo-mixer C, then centrifuged at 3,500 × *g* for 10 min. The resulting supernatant was filtered through a 0.22 µm PTFE syringe filter, and 5 µL of the filtrate was injected into a C18 RP-HPLC column of Hypersil GOLD consisting of particle size of 1.9 µm, dimensions 2.1 mm × 100 mm.

### High-resolution accurate mass spectrometry (HRAMS) based non-targeted metabolite analysis

Reversed-phase chromatographic run began with a highly aqueous mobile phase with formic acid (0.1%) to a highly organic phase of methanol (0.1% formic acid), progressing from aqueous (100%) to organic phase (100%). The LC gradient parameters were as follows: 0–6 min (methanol 5%), 6–10 min (methanol 30%), 10–20 min (methanol 50%), 20–25 min (methanol 90%), 25–27 min (methanol 90%), and 27–30 min (methanol 5%). The flow rate was maintained at 300 µL/min, with the column oven set to 40°C.

The three technical replicates of the respective sample underwent metabolomics analysis. The Thermo Scientific Tribrid HRAMS, “Orbitrap Eclipse,” joined with an Ultra-HPLC (Dionex UltiMate 3000 RSLC) system and a Heated Electrospray Ionization source, was used to project the sample into the MS after separation done by chromatography. The Orbitrap analyzer operated at a resolution of 60,000 for both positive and negative polarities, covering a mass range of *m/z* 100–1,000, with a 35% RF lens and 25% normalized AGC target. The onset of 2.0e5 intensity was set for MS-OT (Master Scan) acquisition. For the ddMS2 OT HCD study, the parameters were quadrupole with a 1.5 *m/z* isolation window, HCD activation type, collision energies (30, 40, and 60) percentage, 15,000 resolution, and normalized AGC (20% target). The raw data obtained from the mass analyzer were handled using the default parameters of “Compound Discoverer 3.3.2.31” and analyzed against online databases. Online databases and mzLogic were used to detect unknowns. The workflow of untargeted metabolomics was: the MetaboAnalyst software accomplishes alignment of retention time, detection of unknown compounds, and grouping of compounds in all samples. For all compounds, the essential compositions were predicted, gaps were filled, and chemical backgrounds were hidden (using Blank samples). Metaboanalyst also recognizes compounds through mzCloud (ddMS2) and ChemSpider (formula or exact mass), and similarity search was achieved for all compounds with ddMS2 data and mzCloud. To rank order ChemSpider results, the mzLogic algorithm was applied. QC-based normalization was performed if QC samples are accessible. The differential analysis (*t*-test or ANOVA) was applied to determine *P* values, adjust *P* values, fold change, and so on. PCA, PLS-DA of metabolites, and generation of heat map were performed by Metaboanalyst (https://www.metaboanalyst.ca/).

### Gene annotation

Gene annotation related to KEGG pathways was conducted using the Integrated Microbial Genomes (IMG) online platform (https://img.jgi.doe.gov/) (69, 70). This platform provides comprehensive genomic data and annotation tools for a wide variety of microbial genomes. The genome of *A. oleivorans* DR1 is publicly available within the IMG database, with complete access to detailed genomic information and pathway analysis. In this study, genes associated with critical metabolic pathways, such as glycolysis/gluconeogenesis, lipid metabolism, biotin synthesis, and benzoate degradation, were specifically identified and annotated. These pathways play essential roles in energy production, cellular growth, and the organism’s ability to degrade complex compounds, which are significant for understanding the metabolic capabilities of *A. oleivorans* DR1.
